# Small-Pox at Purfleet

**Published:** 1903-03-07

**Authors:** 


### SMALL-POX AT PURFLEET.

The very important report upon the incidence of small-pox on the unprotected populations of Purfleet, and of other places in the Orsett Union, which was published last week by the Local Government Board, and which gives a complete account of the inquiries of Dr. G. S. Buchanan into the facts of the case, appears to throw a new light upon the infectivity of the loathsome disease with which it is concerned, and to point to the necessity of new precautions in dealing with it. It will be remembered that Mr. Power, F.R.S., then an inspector of the Board and now the principal medical officer, expressed his belief, many years ago, that small-pox hospitals were sources of danger to the localities in which they were situate, and that infection was distributed throughout these localities by the agency of the atmosphere. Taking a map, on which the position of an urban small-pox hospital was shown and from this position as a centre, Mr. Power described a series of concentric circles, and then placed a dot upon the map for every case of small-pox which was reported to the authorities. He found, quite unmistakably, that cases not traceable to any known source of infection occurred in considerable number within the quarter-mile circle; in diminished number, but still with marked frequency, within the half-mile and three-quarter-mile circles, and that it was not until after the mile boundary had been passed that the disease fell to what might be considered the normal rate for the district. His results excited most attention and inquiry when they were first made public; and although Mr. Power himself did not hesitate to attribute the facts to aerial conveyance of the infection, his explanation was by no means unreservedly accepted. In the country generally, when protests were raised by the inhabitants of any locality against the proposed establishment of a small-pox hospital in their midst, it was usually thought to be a sufficient answer to say that the fear was greater than the danger, and that even small-pox patients must be somewhere.

The continued prevalence and severity of small-pox in Purfleet, and in other places in the Orsett Union, contemporaneously with the occupation of the small-pox ships of the Metropolitan Asylums Board, was in itself almost sufficient to cast doubts upon the correctness of this pleasant optimism; and the inquiry into the facts made by Dr. Buchanan must, we fear, be regarded as its death-blow. The ships are moored close to the Kentish shore, with only the uninhabited Dartford marshes within a mile of them on that side; and they are separated from the Essex shore by the river, which is about half a mile in width. On the Essex shore stands the village of Purfleet, about three-quarters of a mile distant from the ships as the crow flies; and between the ships and the village there is no personal intercourse. During the period from September, 1901, to June, 1902, which is covered by Dr. Buchanan's report, the whole of the Orsett Union suffered severely from small-pox; but the most remarkable feature of the epidemic was its sustained incidence upon Purfleet and the adjoining hamlet of West Thurrock. In Purfleet alone, out of its 600 of civil population, no fewer than 53 contracted the disease, although all the cases were promptly detected, notified, and removed to an isolation hospital. This exceptional severity was not due to a sudden outbreak, but continued without intermission during the 10 months, and was sustained as long as there were any persons in Purfleet who were susceptible to attack. At the end of March the susceptible persons were reduced, by the disease itself and by vaccination and revaccination, to about forty, and fresh cases occurred among these as well as among unprotected newcomers to the locality. Dr. Buchanan also tells us that the circumstances were not peculiar to the recent epidemic, for that whenever small-pox has prevailed in the Union in recent years, since the ships have been occupied, Purfleet and West Thurrock have again and again been the places on which it has earliest fastened. The relationship of these occurrences to the ships can, he says, hardly be doubted; nor can it be questioned that the infection has been conveyed, as Mr. Power long ago suggested it might be, aerially. Additional support is furnished to this view by the circumstance that small-pox appeared during the epidemic among the crews of barges which had anchored in Long Reach a fortnight before, and was rife among workmen employed in erecting temporary buildings for the Metropolitan Asylums Board on the Dartford marshes, within a quarter of a mile of the ships. The conclusion seems to be inevitable that small-pox cannot in any way be rendered other than a public danger, and that the duty of preventing its occurrence is a binding one upon all communities. How easily this may be done is once more shown by the fact that the training ship *Cornwall* was moored within half a mile of the small-pox ships, and that not a single case of small-pox occurred among her 266 inhabitants, or among the 215 persons of the Purfleet garrison, all of whom had been vaccinated and revaccinated.

